# Neuromelanin Magnetic Resonance Imaging Reveals Increased Dopaminergic Neuron Activity in the Substantia Nigra of Patients with Schizophrenia

**DOI:** 10.1371/journal.pone.0104619

**Published:** 2014-08-11

**Authors:** Yoshiyuki Watanabe, Hisashi Tanaka, Akio Tsukabe, Yuki Kunitomi, Mitsuo Nishizawa, Ryota Hashimoto, Hidenaga Yamamori, Michiko Fujimoto, Masaki Fukunaga, Noriyuki Tomiyama

**Affiliations:** 1 Diagnostic and Interventional Radiology, Osaka University Graduate School of Medicine, Suita, Japan; 2 Research Center for Children’s Mental Development, United Graduate School of Child Development, Osaka University, Suita, Japan; 3 Departments of Biofunctional Imaging and Immunology, Frontier Research Center, Osaka University, Suita, Japan; 4 Department of Psychiatry, Osaka University Graduate School of Medicine, Suita, Japan; Duke-NUS Graduate Medical School, Singapore

## Abstract

**Purpose:**

The dopamine hypothesis suggests that excessive dopamine release results in the symptoms of schizophrenia. The purpose of this study was to elucidate the dopaminergic and noradrenergic neurons using 3-T neuromelanin magnetic resonance imaging (MRI) in patients with schizophrenia and healthy control subjects.

**Methods:**

We prospectively examined 52 patients with schizophrenia (M: F = 27∶25, mean age, 35 years) and age- and sex-matched healthy controls. Using a 3T MRI unit, we obtained oblique T1-weighted axial images perpendicular to the brainstem. We measured the signal intensity and area for the substantia nigra (SNc), midbrain tegmentum, locus ceruleus (LC), and pons. We then calculated the contrast ratios (CR) for the SNc (CR_SN_) and LC (CR_LC_), which were compared between patients and healthy controls using unpaired *t*-tests.

**Results:**

The SNc and LC were readily identified in both patients and healthy controls as areas with high signal intensities in the posterior part of the cerebral peduncle and in the upper pontine tegmentum. The CR_SN_ values in patients were significantly higher than those in healthy controls (10.89±2.37 vs. 9.6±2.36, p<0.01). We observed no difference in the CR_LC_ values between the patients and healthy controls (14.21±3.5 vs. 13.44±3.37, p = 0.25). Furthermore, there was no difference in area of the SNc and LC between schizophrenia patients and controls.

**Conclusions:**

Neuromelanin MRI might reveal increased signal intensity in the SNc of patients with schizophrenia. Our results indicate the presence of excessive dopamine products in the SNc of these patients.

## Introduction

Dopamine dysfunction plays an important role in the pathogenesis of schizophrenia [1]. The dopamine hypothesis suggests that excessive dopamine release results in symptoms of schizophrenia. *In vivo* positron emission tomography (PET) studies in patients with schizophrenia have indicated an increased baseline occupancy of D2 receptors by dopamine [2] and an increased capacity for striatal dopamine synthesis [3]. However, PET is not widely available, and its use in research is limited because of its high production costs. Further, the short half-life of ^11^C radiopharmaceuticals restricts their use to only institutions having a cyclotron on-site.

Neuromelanin is a byproduct of the synthesis of monoamine neurotransmitters, such as noradrenalin and dopamine, and is mainly distributed within neurons of the substantia nigra (SNc) or locus ceruleus (LC) [4]. Neuromelanin has a T1-shortening effect, which was a similar characteristic of the cutaneous melanin. High-field magnetic resonance imaging (MRI), such as 3 T, is very sensitive to tissue T1 relaxation and are able to depict tissue containing neuromelanin in (SNc) or (LC) [5]. There are many previous reports which showed the signal decrease in Parkinson’s disease using neuromelanin MRI [4], [6]–[10], but there are only two reports using this technique for schizophrenia [11], [12].

The purpose of this study was to use 3T neuromelanin MRI for examining dopaminergic and noradrenergic nuclei in patients with schizophrenia and healthy controls.

## Materials and Methods

### Subjects

From April to November 2012, we prospectively examined 63 consecutive patients with schizophrenia who met the *Diagnostic and Statistical Manual of Mental Disorders, 4^th^ Edition*, (DSM-IV) diagnostic criteria using 3T-MRI. Eleven patients were excluded because of motion artifacts (6 patients) and equipment failure (5 patients). Therefore, 52 patients (M: F = 27∶25; mean age = 35 years; range = 17–69 years) were included in the analysis. In addition, we obtained MRI data from age- and sex-matched healthy controls. Controls were recruited from the community through local advertisements at Osaka University. An institutional review board approved this study, and written informed consent was obtained from all subjects before their participation. We used the Japanese version of the Positive and Negative Symptom Scale (PANSS) [13] to assess patient symptoms and their severity scores. We administered the Japanese version of Wechsler Adult Intelligence Scale III [14] to determine the full scale intelligence quotient (IQ). Premorbid IQ was estimated using the Japanese Adult Reading Test [15], [16].

This study was performed in accordance with the World Medical Association’s Declaration of Helsinki and approved by the local institutional review board (2013-423, Osaka University Ethics Committee). Written informed consent was obtained by all subjects. If the subjects were under 20 years old, written informed consent was obtained from both minors and guardians. If the patients with schizophrenia were difficult condition to accept consent by theirself, these patients were not included in this study.

### Imaging protocol

Using a 3T MRI unit (Signa Excite HDxt, GE healthcare, Milwaukee, Wisconsin), we obtained oblique T1-weighted oblique axial images perpendicular to the brainstem. The T1-weighted sequence was acquired with a 3D-spoiled GRASS sequence with magnetization transfer contrast: TR/TE = 38.4/2.4 ms, FA = 20 degrees, matrix size 480×320 in axial plane, FOV = 220 mm, and acquisition time = 3 min 25 s. A 40-mm slab thickness was used and images were reconstructed 40 slices with a slice thickness of 2 mm with in-slice zero-fill interpolation (ZIP2). We also obtained axial T2-weighted images of the whole brain to exclude coexisting disorders and any abnormal findings that might influence the signals for the SNc or LC. The T2-weighted image parameters are as follows: TR/TE = 4500/88 ms, FOV = 220 mm, Matrix = 512×256, 24 slices with slice thickness 5 mm, and 6 mm slice interval.

### Data analysis

**Figure 1:**
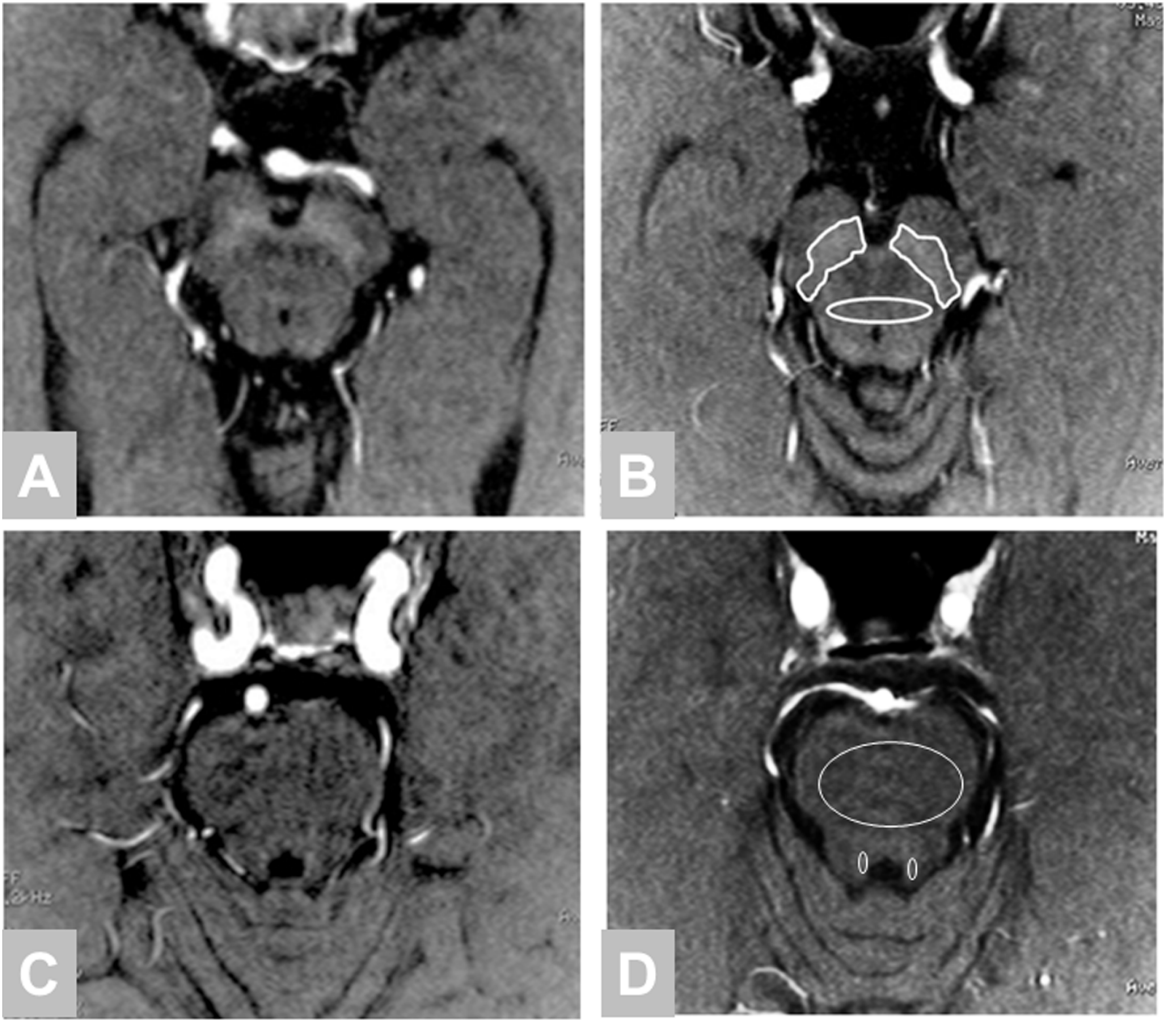
Neuromelanin imaging in the midbrain. A, C: 30-year-old male with schizophrenia, CR_SN_ = 12.9, CR_LC_ = 10.7; B, D: 26-year-old female healthy control, CR_SN_ = 6.2, CR_LC_ = 7.6 Demonstrating a region of interest drawn around the substantia nigra and midbrain tegmentum side on Figure 1B and locus ceruleus on Figure 1D.

We measured the signal intensity of the SNc, midbrain tegmentum, LC, and pons. The region of interest (ROI) for the SNc was traced manually around the high signal area on two consecutive axial slices and ellipse ROI was set at midbrain tegmentum in the same slice (Figure 1B). An ellipse ROI for the LC and pons were indicated on three consecutive slices. The average and maximum signal intensities (MaxSR) and area were measured for each ROI. The measurements were performed by a blinded author. We calculated the contrast ratio (CR) of the SNc (CR_SN_) and LC (CR_LC_) using the following equations: CR_SN_ = (S_SN_−S_TM_)/S_TM_, CR_LC_ = (S_LC_−S_P_)/S_P_. In these equations, S_SN_ and S_TM_ are the signal intensities for the SNc and midbrain tegmentum, respectively, and S_LC_ and S_P_ are the signal intensities of the LC and pons, respectively.

To reduce the effects of age-related changes in the CR_SN_ and CR_LC_, we selected a subset of subjects who were under 30 years of age (n = 24 for schizophrenia, n = 29 for healthy controls) and compared their ROI values.

Statistical analyses were performed using unpaired *t*-tests to determine the differences between patients with schizophrenia and healthy controls.

We calculated the correlation between age and CR_SN_ or CR_LC_ for patients with schizophrenia and healthy controls. To elucidate the medication effects for the contrast ratio, the correlation between the chlorpromazine (CPZ) equivalents and CR_SN_ or CR_LC_ was analyzed.

## Results

Table 1 presents the characteristics and clinical symptoms of patients and healthy controls. Compared to the patients, healthy controls had more years of education and higher IQs.

**Table 1:** Clinical characteristics of patients with schizophrenia and healthy controls.

	Schizophrenia	Control		Schizophrenia	Control	
	all n = 52	all n = 52	p value	<30 year n = 24	<30 year n = 29	p value
Age	35.1 (13.3)	34.6 (13.7)	0.89	23.8 (4.1)	23.0 (2.3)	0.34
Sex (male:female)	27∶25	27∶25		11∶13	16∶13	
Year of education	13.4 (2.5)	15.4 (2.1)	<.001	13.0 (2.7)	15.6 (1.6)	<.001
Smoking (%)	16 (31%)	4 (7.7%)	<.001	6 (25%)	1 (3.4%)	<.001
Estimated premorbid IQ.	102.0 (10.9)	109.7 (7.3)	<.001	102.0 (11.2)	111.3 (5.0)	<.001
Full scale IQ	87.0 (20.9)	113.8 (14.1)	<.001	87.8 (20.4)	118.4 (11.4)	<.001
Age of onset	22.9 (10.1)			18.2 (3.3)		
Duration (years)	10.4 (10.9)			4.9 (4.6)		
CPZeq (mg/day)	596.2 (556.2)			495.8 (541.6)		
PANSS positive	21.0 (6.3)			18.3 (6.3)		
PANSS negative	23.1 (7.5)			20.1 (6.4)		
PANSS general	50.0 (13.9)			45.8 (13.6)		
PANSS total	94.1 (26.2)			84.1 (25.5)		

Data are shown mean (standard deviation). CPZeq: chlorpromazine equivalent of total antipsychotics.

IQ: Intelligence Quotient, PANSS: Positive and Negative Symptom Scale.

**Figure 2:**
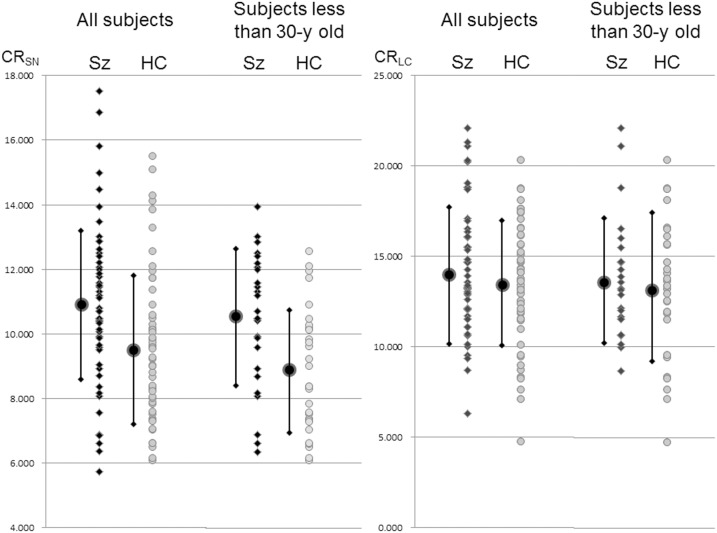
CR_SN_ and CR_LC_ for patients with schizophrenia and healthy controls. The left graph plots data for all subjects. The right graph plots data for selected patients under 30 years of age. The dots and bar show mean ± standard deviation. The patients showed a significantly higher CR_SN_, but the variation in each group was large. The dispersion of data is small to select the young patients. There was no significant difference about CR_LC_ between the patients and healthy controls.

The SNc and LC were readily identified by high signal intensity areas in the posterior part of the cerebral peduncle and at the upper pontine tegmentum in both patients and healthy controls (Figure 1). Table 2 summarizes the mean signal intensities of each ROI. Our quantitative analysis showed that the CR_SN_ values and MaxSR SNc were significantly higher in patients with schizophrenia than in healthy controls (CR_SN_: 10.89±2.37 vs. 9.6±2.36; p<0.01; Figure 2; MaxSR SNc: 1.32±0.04 vs. 1.30±0.04; p<0.05). No difference was observed in the CR_LC_ values between the patients and healthy controls (14.21±3.5 vs. 13.44±3.37; p = 0.25; Figure 2). There was no difference the areas of the SNc and LC between schizophrenia and healthy controls.

**Table 2:** Neuromelanin imaging characteristics of patients with schizophrenia and healthy controls.

	Schizophrenia	controls	Schizophrenia	controls	Schizophrenia	controls
	all age n = 52	all age n = 52	<30 years n = 24	<30 years n = 29	≧30 year n = 28	≧30 year n = 23
CR_SN_ (%)	10.89±2.37*	9.60±2.36	10.51±2.11$	8.85±1.95	11.22±2.80	10.55±2.51
CR_LC_ (%)	14.21±3.5	13.44±3.37	13.73±3.37	13.15±3.88	14.63±3.56	13.79±2.69
Area-SNc (mm^2^)	160.1±24.1	162.2±21.6	155.4±16.9	164.8±24.7	164.2±28.1	158.9±17.1
Area-LC (mm^2^)	10.84±2.46	11.42±2.27	10.79±1.93	11.67±2.57	10.89±2.82	11.09±1.86
MaxSR SNc	1.32±0.04*	1.30±0.04	1.30±0.03*	1.28±0.03	1.33±0.04	1.32±0.04
MaxSR LC	1.28±0.05	1.27±0.05	1.27±0.05	1.28±0.06	1.29±0.05	1.27±0.04

SR, signal ratio; SNc, substantia nigra; LC, locus coeruleus; CR_SN_, contrast ratio of SNc; CR_LC_, contrast ratio of LC;

MaxSR, maximum signal intensity.

Data are presented as mean ± standard deviation.

*p<0.05 compared to controls,

$ p<0.005 compared to controls.

In the subset of subjects that were under the age of 30, CR_SN_ values were significantly higher in patients with schizophrenia than in healthy controls (10.51±2.11 vs. 8.85±1.95; p<0.005; Figure 2). There was no significant difference for the subset of subjects over 30 years old.

There is weak correlation between age and CR_SN_ (R = 0.325, p = 0.019) for healthy controls and (R = 0.263, p = 0.053) for schizophrenia. There is no correlation between age and CR_LC_ (R = −0.008, p = 0.95) for healthy controls and (R = 0.196, p = 0.164) for schizophrenia. The CPZ equivalent and CR_SN_ showed weak correlation (R = 0.353, p = 0.010) and there is no correlation between CPZ equivalent and CR_LC_ (R = 0.023, p = 0.870) for patients with schizophrenia.

## Discussion

Our results demonstrate the excessive levels of dopamine products in the SNc of living patients with schizophrenia and this supports the dopamine hypothesis for schizophrenia. Recently, Howers et al [17] reported the same results using a post-mortem study, which revealed that tyrosine hydroxylase staining scores were significantly greater in the schizophrenia group at substantia nigra compared to in healthy controls and in vivo imaging using PET which showed that elevated dopamine synthesis was seen in the nigral dorpamine neurons in schizophrenia.

It has been suggested that dopamine dysfunction plays an important role in the pathogenesis of schizophrenia. This hypothesis is supported by evidence provided by numerous observations and studies. For example, the stimulants amphetamine and cocaine, which increase dopamine levels in the brain, can cause symptoms resembling those for psychosis [18]. Patients with Parkinson’s disease who have been treated with levodopa, a dopamine-enhancing compound, can experience psychotic adverse effects mimicking the symptoms of schizophrenia [19]. Antipsychotic drugs such as chlorpromazine, however, can antagonize dopamine D2 receptor binding and reduce the positive symptoms of psychosis [20]. Lots of in vivo studies have used PET techniques that examine receptor imaging or dopamine synthesis in order to evaluate the dopamine system in patients with schizophrenia. Dopamine D2 receptors were upregulated in patients with schizophrenia [21] and increased striatal dopamine synthesis occurs in schizophrenia [3]. However, because of low-resolution of PET, many studies evaluated at striatum and cerebral cortex.

It has been reported that T1-weighted MRI with 3T can indicate T1-shortening tissues containing neuromelanin at SNc and LC [4], [5]. This technique is widely used to investigate neuromelanin signal and volume loss in the SNc of patients with Parkinson’s disease [6]–[9], [22]. We are aware of only two previous reports on neuromelanin imaging in patients with schizophrenia. Shibata et al [11] described signal changes in the SNc and LC among patients with schizophrenia, depression and controls. However, the CR_SN_ values were higher in patients with schizophrenia (*n* = 20; 22.6±5.6: mean ± standard deviation (SD)) than in those with depression (*n = *18; 19.2±4.7), as well as controls (*n = *34; 19.6±3.8; one-way ANOVA, *p* = 0.037). However, a post hoc Tukey’s test indicated no significant difference among schizophrenia and controls.

Sasaki et al [12] reported the CR_SN_ values were higher in patients with schizophrenia (*n* = 23; 22.6±5.1) than those with depression (*n* = 23; 19.0±4.3) and controls (*n* = 23; 20.5±3.4). A post hoc test confirmed a significant difference between patients with schizophrenia and those with depression, but not between patients with schizophrenia and controls.

These two previous reports indicate the CR_SN_ in patients with schizophrenia is higher than that in controls, but this was not statistically significant due to a small sample size and large variations. We performed a similar comparison in our study using a lager patient group (52 patients) and observed a significant difference. The mean CR_SN_ was significantly higher in patients with schizophrenia than in healthy controls, but the CR_SN_ values showed large variations with overlap between patients and healthy controls (Figure 2). It is reported that neuromelanin levels in the SNc can increase with age using post-mortem histrogical examination [23], [24] and also neuro-melanin MRI [23], [25]. The age range in our study was very broad, ranging from 17 to 69 years. To reduce age-related changes, we selected subjects younger than 30 years. Between these groups, the CR_SN_ value showed small variations, and the difference between patients and healthy controls was more prominent (Figure 2).

The absolute value of CR_SN_ observed in our study differed from those in two previous reports, which likely resulted from differences of the reference ROIs and MR sequence. We used a reference ROI located in the midbrain tegmentum, whereas previous reports used the decussation of the superior cerebellar peduncle. We use 3D-SPGR sequence to obtain T1-weighted image and previous reports performed a 2D-fast spin echo sequence. 3D acquisition is superior to obtain high signal to noise ratio image in less time. We showed significant difference using short time neuromelanin imaging and this was of great advantage to exam the schizophrenic patients who were sometimes difficult to hold steady head position for long examination time in MR unit.

There are several limitations to our study. First, we included all patients examined with neuromelanin MRI from April to November 2012. Many of these patients were under treatment with antipsychotic drugs. Our results showed that there is a weak correlation between CPZ equivalent and CR_SN_, therefor these drugs have the potential to influence neuromelanin levels. The number of participants in this study was not enough to perform subgroup analysis and further study is needed to select first episode medication-naive patients to exclude the drug effect. Second, we could not evaluate the ventral tegmental area (VTA), which is the origin of the dopaminergic cell bodies of the mesolimbic-cortical dopaminergic system, because of the difficulty in detection the border of the VTA by neuromelanin-MRI. Third, the years of education and IQ were significantly higher in healthy controls than in patients with schizophrenia. Schizophrenia is characterized by general intellectual deficits [26], [27]. The estimated premorbid IQ difference between schizophrenia and normal controls is smaller compared to that of full scale IQ, but the difference is statistically significant [28], [29]. Fourth, we used 3D-SPGR imaging for neuromelanin MRI and the acquisition time was 3 min 25 s. Our acquisition time is shorter than that used for 2D-fast spin echo sequences in previous studies [4], [5], [11], [12], [30] and this short acquisition might make lower signal to noise ratio and large signal variability.

In conclusion, neuromelanin MRI revealed increased signal intensity in the SNc of patients with schizophrenia. This finding indicates the presence of an excessive level of dopamine products in the SNc of these patients. Therefore, neuromelanin imaging has the potential to be useful for accurate diagnosis of schizophrenia and to serve as a surrogate marker for medication.
